# A Novel Smartphone App to Support Learning and Maintaining Competency With Bier Blocks for Pediatric Forearm Fracture Reductions: Protocol for a Mixed-Methods Study

**DOI:** 10.2196/10363

**Published:** 2018-12-21

**Authors:** Brett Burstein, Adam Bretholz

**Affiliations:** 1 Division of Pediatric Emergency Medicine Montreal Children's Hospital McGill University Health Centre Montreal, QC Canada

**Keywords:** intravenous regional anesthesia, lidocaine, procedural sedation, mobile phone

## Abstract

**Background:**

Distal forearm fractures are among the most common injuries presenting to the pediatric emergency department (PED). Bier block (BB), or intravenous regional anesthesia, is a safe and effective alternative to procedural sedation for closed reduction of forearm fractures; it is associated with fewer adverse events, a shorter length of stay, and reduced costs. BB has long remained relatively underutilized; however, with an increasing emphasis on efficient resource use and patient-centered care, there is renewed interest in this technique.

**Objective:**

Our tertiary PED recently became the first in Canada to successfully implement an active BB program. Subsequently, we developed a mobile BB smartphone app designed to support the sustained departmental use of BB. The app can be used for training and maintenance of competency and incorporates instructional material, as well as our institutional BB protocol, printable medication order sheets, and monitoring forms. The present report describes the development and functionality of the BB smartphone app.

**Methods:**

We have described app development and content. App dissemination metrics will be tracked, and user feedback will be analyzed using a self-administered electronic survey. Additionally, app utilization in our PED will be compared with real-world clinical use of BB for fracture reductions.

**Results:**

The first iteration of the BB app was launched in 2015, with the most recent update in September 2018. App metric tracking is planned for January 2020 until December 2021.

**Conclusions:**

We have highlighted how the BB app serves as a paradigm of an educational tool designed not only for individual users but also for supporting the department-wide implementation and dissemination of a new technique. App dissemination and use metrics will be tracked and correlated with clinical use of BB in the PED.

**International Registered Report Identifier (IRRID):**

PRR1-10.2196/10363

## Introduction

### Background

Bier block (BB), or intravenous regional anesthesia, is a safe and effective alternative to procedural sedation for forearm fracture reduction [1-3]. Using a pneumatic tourniquet, the isolated injured extremity is injected with local anesthetic to provide complete limb analgesia below the tourniquet cuff for painless fracture reduction. The patient remains conscious throughout the procedure, for which, therefore, no preprocedural fasting is required, postprocedure observation period is minimal, and potential risks of procedural sedation are avoided. Intravenous access is required only on the affected limb and only briefly during infusion of local anesthetic. Consequently, this technique is associated with reduced length of stay and resource utilization [4] and can be used for children as young as 2-4 years [1,2,4].

Despite BB’s apparent benefits, it remains infrequently employed for forearm fracture reductions. A survey of 44 North American pediatric emergency departments (PEDs) reported that the most common reason cited for not using local anesthesia techniques was the efficacy of procedural sedation. The authors further suggested that limited physician comfort and perceived longer preparation time were important barriers to BB use [5]. These factors, coupled with sporadic clinical opportunity, departmental logistics, and personnel turnover, are all important challenges to the successful implementation of a sustained BB program.

Recently, our center became the first Canadian PED to successfully introduce a BB program for forearm fracture reductions. PED personnel were trained at the time of BB implementation using a multimodal training course. We observed a sustained increase in BB utilization following training, reaching nearly 40% of all forearm fracture reductions at 2 years. However, course participants reported a modest but significant decrease in comfort with BB at 6 months, and the majority of participants expressed interest in refresher training [6].

### Objectives

Smartphones are widely used and medical smartphone apps have been shown to support the acquisition of technical skills [7]. We have developed a novel point-of-care smartphone app designed to facilitate learning and maintenance of BB competency. Here we report on the app development process and functionality, with an emphasis on both its utility for individuals interested in using BB and its function as a model for the development of an educational tool to support the implementation of any new technique in the emergency department (ED). App dissemination metrics will be tracked, and its utilization at our center will be compared with real-world clinical use of BB for fracture reductions.

## Methods

### Mobile App Content and Functionality

**Figure 1:**
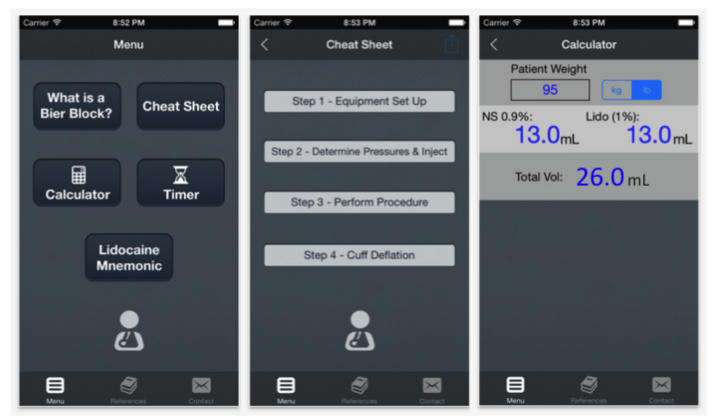
Main menu and selected screenshots of the BB smartphone app. (Source: Apple App Store Store, MUHC Bier Block App; Developer: Silverbirch Ventures, Toronto, ON).

The app functions as a self-contained tool to support the utilization of BB by both physicians and nurses and may be used by either novice or experienced users. Additionally, the app contains supporting resources necessary to facilitate the establishment of a departmental BB program. The BB app (Figure 1) contains a brief description of BB and a demonstration video. From the main screen, users can find complete step-by-step instructions. A dose calculator determines weight-based lidocaine dosing, in metric or imperial units with maximal dosages, and a timer directly integrates a stopwatch function. A user-friendly memory aid is provided for the signs, symptoms, and management of lidocaine toxicity. A tab from the main menu links to evidence-based BB references including our institutional protocols for BB and local anesthetic toxicity management, a medication order sheet with integrated dosage calculator, and a safety monitoring record. All reference materials can be saved, printed, or emailed from within the app directly. All content was adapted from our evidence-based institutional BB training course [6].

Feedback was collected from departmental stakeholders following BB training to determine preferences for app content and features. The app was piloted for technical ease of use and clarity, and ongoing quality feedback was incorporated into subsequent app updates. All content and protocols received institutional approval for release from the McGill University Health Centre.

### Technical Requirements and Associated Costs

Beta testing was performed using Apple’s proprietary TestfFight software (Apple Inc). Total app storage requirement is 30 MB, and once installed on a mobile device, all content is available without internet connection. The current version is compatible with Apple iOS devices and is available for free through the Apple App Store. Development costs were under Can $1250 in addition to a mandatory fixed recurring cost of Can $100 per year to maintain an Apple developer account.

### App Evaluation

App dissemination will be tracked using the analytics provided by the app builder and the Apple App Store Analytics software, as described elsewhere [8]. Metrics tracked will include Web-based app views, referrals, user engagement (number of sessions and active devices), total downloads, and retention. Users will be invited via the app to participate in a self-administered electronic survey to assess self-reported perceptions of the BB app. User-perspective surveys will be developed and reported according to published guidelines [9]. Specific data on app utilization and timing of use will be tracked among users in our PED in parallel to real-world clinical use of BB for pediatric fracture reductions [6]. Tracking of app usage in our PED will make use of the external tester function of Apple TestFlight software.

## Results

The BB mobile app was launched in January 2015, with updates incorporating user feedback until 2016, and underwent a final content update in September 2018. After receiving approval by the McGill University Health Centre Research Ethics Board, app analytics and clinical correlation metrics will be evaluated between January 2020 and December 2021.

## Discussion

The BB app was developed to improve and maintain comfort of individuals performing BB and to enhance the sustainable functioning of a departmental BB program. The app was designed for interdisciplinary use by both nurses and physicians. This point-of-care tool supports maintenance of competency that is often limited by sporadic clinical opportunities when used alone as a quick reference, in combination with other models’ procedural learning such as simulation, or as a cognitive aid during both simulation and real-world use. To our knowledge, this is the only app for BB, and it is unique in its range of functionality.

Despite the rapid proliferation of medical smartphone apps, there is a paucity of literature on the use of apps to learn or support ED procedures, and such apps are very few. The majority of currently available medical apps contain basic demonstration videos, instructional texts, clinical decision rules, or calculators. More recently, apps with greater functionality and sophistication have been described for point-of-care use in acute care settings [10]. Hawkes et al have described an app for neonatal intubation that increased physician knowledge and improved procedural performance [7].

The BB app is also exemplary of how a smartphone tool can complement and augment the introduction of a new technique throughout a department or institution. Procedure preparation time is shortened by convenient access to printable medical order sheets, monitoring forms, and dose calculators. The use of such an app may reduce the time and resources required for frequent departmental refresher training and can help bridge the gap between courses for infrequent users or new personnel. Moreover, full evidence-based protocols within the app facilitate knowledge translation to other departments and institutions wishing to implement a similar program.

Our BB app does not provide details regarding pneumatic cuff inflation and safety checks, which vary by make and model of the automatic tourniquet system. Importantly, the BB app itself has not undergone peer review; however, this is not unusual or unique to our app [11], but could someday become necessary in a changing regulatory environment [12]. Moreover, we have not quantified the effect of the app to prevent the attrition of comfort among BB users and how the app compares to refresher courses. Understanding how to best integrate this and other apps into current educational models of procedural learning remains to be studied, but is beyond the scope of this report.

In summary, BB is safe and effective for pediatric forearm fracture reductions but remains underutilized. We have developed a novel BB smartphone app to overcome the barriers to more widespread use of this technique. The app is not only a resource for individual BB users but also provides a case study for how an institution can develop an educational tool to support a new program and enhance knowledge translation with other centers.

## Abbreviations

BB: Bier block
ED: emergency department
PED: pediatric emergency department
